# Relationship between CK19 expression, deregulation of normal keratinocyte differentiation pattern and high risk-human papilloma virus infection in oral and oropharyngeal squamous cell carcinoma

**DOI:** 10.1186/s13027-015-0041-x

**Published:** 2015-12-15

**Authors:** Angela Santoro, Giuseppe Pannone, Rossella Ninivaggi, Massimo Petruzzi, Andrea Santarelli, Giuseppe Maria Russo, Silvia Lepore, Michele Pietrafesa, Ilaria Laurenzana, Rosalia Leonardi, Paolo Bucci, Maria Iole Natalicchio, Alberta Lucchese, Silvana Papagerakis, Pantaleo Bufo

**Affiliations:** Department of Laboratory and Services, Institute of Histopathology and Diagnostic Cytopathology, Fondazione di Ricerca e Cura ‘Giovanni Paolo II’-UCSC, Largo Agostino Gemelli, 1, Campobasso, Italy; Department of Clinical and Experimental Medicine, Institute of Pathological Anatomy, University of Foggia, Foggia, Italy; Department of Odontostomatology and Surgery, University of Bari, Bari, Italy; Department of Clinic Specialistic and Stomatological Sciences, Polytechnic University of Marche, Ancona, Italy; Laboratory of Preclinical and Translational Research, IRCCS-CROB, Oncological Reference Centre of Basilicata, Rionero in Vulture, Italy; Department Medical-Surgical Specialties, Section of Oral Medicine, Policlinico, University of Catania, Catania, Italy; Department of Neurosciences, Reproductive and Odontostomatological Sciences, Institute of Oral Pathology, University of Napoli ‘Federico II’, Naples, Italy; Molecular Biology Laboratory, Azienda Ospedali Riuniti, Foggia, Italy; Multidisciplinary Department of Medical-Surgical and Odontostomatological Specialties - Second University of Napoli (SUN), Naples, Italy; Department of Otolaryngology - Head and Neck Oncology, University of Michigan, 500 S State St, Ann Arbor, MI 48109 USA

**Keywords:** HR-HPV, Immunohistochemistry, Cytokeratins

## Abstract

**Background:**

Simple epithelial keratins appear early during embryonic development and are expressed in non-stratified, ductal and pseudo-stratified epithelial tissues. CK19, the lowest molecular weight keratin, is also expressed in basal layer of squamous epithelia of mucosal surfaces. Previous studies have shown that High Risk-Human Papilloma Virus (HR-HPV) epithelial infection induces cell immortalization via E6 and E7 viral proteins and this, in turn, impairs cytokeratin expression in cancerous cells lines derived from uterine cervix. Here, we demonstrate the possible relationship between HR-HPV^+^ oral/oropharyngeal cancer and the high levels of CK19 expression.

**Methods:**

We analyzed 38 cases of Oral Squamous Cell Carcinomas/ Oro-Pharyngeal Squamous Cell Carcinomas (OSCCs/OPSCCs) by Immunohistochemistry (IHC) using specific antibody (Ab) detecting CK19, by In Situ Hybridization (ISH) and Polymerase Chain Reaction (PCR) based methods in order to define the HPV infectious status. We also evaluated the variation of CK19 expression in UPCI-SCC-131 (HPV^−^) and UPCI-SCC-154 (HPV^+^) cell lines by immunocytochemistry (ICC) and flow cytometry.

**Results:**

CK19 OSCC/OPSCC score has been identified multiplying percentage of cancer expressing cells to staining intensity. CK19 expression score in OSCCs/OPSCCs was very different between HPV^+^ (mean: 288.0 ± 24.3) and HPV^−^ cancers (mean: 66.2 ± 96.9). This difference was statistically significant (*p* < 0.001) with a strong evidence of correlation (*p* < 0.001; Spearman’s R: +0.72). ROC curve analysis was performed on CK19 expression index related to HPV positivity. Heterogeneous areas of immunoreactivity varying in percentage value, intensity and/or localization were observed in normal epithelium, both perilesional and distant from the tumor with important differences between HR-HPV^+^ and HR-HPV^−^ carcinomas. By ICC and flow cytometry, the two analyzed cell lines were both CK19 positive but showed a different level of expression, in particular it should be noted that the UPCI-SCC-154 (HPV^+^) cell line had a higher expression than UPCI-SCC-131 (HPV^−^).

**Conclusions:**

In this study we demonstrated, for the first time, strong association between CK19 up-regulation and HR-HPV^+^ OSCCs/OPSCCs. This test has a good accuracy. We identified ROC curve with a cut-off > 195 for HR-HPV positive results (Sensitivity: 92.3 %; Specificity: 89.3 %). Furthermore, in OSCC/OPSCC, the CK19 test may be useful in identifying HR-HPV infection, the latter being related to HPV E7 potential to disrupt normal cytokeratin expression pattern.

## Introduction

According to epidemiological studies, a real shift in OSCCs/OPSCCs aetiology may be one of the main reasons of the recent reported improvements in survival of a specific subgroup of patients, treated with radiotherapy [1]. From the first report of Syrjanen et al. in 1983 [2], several other Authors have identified HR-HPV in Head and Neck cancers and confirmed the causal role of HPV in a sub-group of oral-oropharyngeal cancers [3–7].

According to Bernard et al. the heterogeneous Papillomaviridae family now contains 29 genera formed by 189 papillomavirus types, isolated from humans, non-human mammals, birds and reptiles. In particular 120 fully sequenced genotypes have been isolated from humans [8]. As a large group of host specific DNA virus, HPVs are characterized by a considerable broad epithelial cell tropism. Considering their potential risk to induce an invasive cancer, almost 45 subtypes, isolated from the low genital tract, have been all along grouped into high- and low- risk HPV types [9]. Munoz et al. classified HPVs in three oncogenic types: high risk viruses (HPV16, 18, 31, 33, 35, 39, 45, 51, 52, 56, 58, 59, 68, 73 and 82), potential high risk viruses with a not well known oncogenic potential (HPV26, 53 and 66), and viruses with low oncogenic risk (types 6, 11, 40, 42, 43, 44, 54, 61, 70, 72, 81 and 89) [10]. More recently, the IARC Working Group has defined a carcinogenic role for the HPV types 16, 18, 31, 33, 35, 39, 45, 51, 52, 56, 58, 59 and a probably carcinogenic potential for the type 68 [11, 12]. Meta-analyses have proved that HPV subtypes associated with head and neck squamous cell cancer (HNSCCs) are broadly similar (but not completely identical) with those classically observed in cervical carcinoma [13, 14]. This is likely to reflect not only a difference in viral life cycles in various and distant mucosal locations, but also an associated diversity in mucosal local immune responses [10, 15]. On the whole, HPV16, the most common high risk HPV detected in cervical squamous cell cancer (50-60 %), was also the most common type detected in HNSCCs (85-95 %) [16].

HPVs exert their oncogenic potential by expressing E6 and E7 viral proteins in infected host cells.

These proteins affect cell cycle in terms of proliferation induction and deregulation by targeting, respectively, p53 and pRB tumor suppressors, up to promote carcinogenesis. In particular, it appears plausible that E7 oncogene is able to redirect terminally differentiating epithelial cells to support viral DNA amplification, and in this way the generation and the maintenance of progeny virus [17].

Integration of HPV DNA into the host DNA, as key-mechanism to induce high grade lesions and invasive cancer [18, 19], is a topic well known in cervical carcinoma but with only few investigations in Head and Neck cancers and numerous points of discussion. According to recent observations, in oropharyngel cancers HPV is almost exclusively not integrated and its carcinogenic activity is due to E6/E7 oncoprotein expressed from episomal viral status [20, 21].

The exact molecular mechanisms used by HPVs to control the replication of their own genome and to promote and preserve their transcriptional activity to the detriment of host infected cell are extremely tangled. Although the viral mRNA translation process is intricately tied to the differentiation program of the host epithelial tissue, it is broadly acknowledged that the expressed viral oncoproteins are highly able to induce keratinocytes immortalization and to enhance the disruption of the normal cytokeratin (CK) expression pattern in stratified squamous epithelium [22], in this way favoring the stepwise process that leads to the onset of squamous cell carcinoma. It has postulated that the preferred target of HPV is the basal layer of the mucosal epithelium. In this germinal layer, the interaction HPV-DNA and host cell promotes cellular proliferation, radical change in cellular metabolism and deregulation in the production of cytokeratins. For example, in HPV infected uterine cervix some Authors have reported deviations in the expression of high molecular weight cytokeratins (HMW-CKs) [23, 24].

Cytokeratins are proteins containing 10 nm intermediate filaments found in the intracytoplasmic cytoskeleton of epithelial tissue, with a molecular weight of 40-68 kDa [25]. They set up a complex family of proteins, including at least 19 cytokeratins, divided into low versus high molecular weight (LMW-CKs vs HMW-CKs) solely based on their molecular weight or according to their acidity and alkalinity in two specific types: the acidic type I (including CK9-23) and the basic type II (including CK1-8) [26, 27]. Expression of these cytokeratins depends mainly on the epithelium type and on the status of epithelial terminal differentiation and maturation. By applying this concept also to the malignant epithelial counterparts, in surgical pathology keratin IHC is considered a tool of immense value widely used to assess cancer differentiation and characterization.

According to the reported literature, normal oral mucosa expresses cytokeratins 4, 5, 13, and 14 [28]. This pattern seems to be kept or very similar to the normal in benign epithelial lesions, while it is resulted irregular and markedly altered in malignant tumors [29].

An early study by Kellokoski J, et al. evaluated by IHC the distribution of cytokeratins 19, 14, 16 and 17 and 8 and 18 in 96 oral mucosal biopsies taken from women with genital HPV infections. They observed a more efficient and intense CK19 expression in the basal cell layer of HPV DNA-positive samples, suggesting for the first time that viral infection disturbs the keratinocyte differentiation in the basal cells of oral epithelium accelerating the production of LMW cytoskeletal protein [30].

Up to now analyses of the specific epithelial keratin profile in HPV infected oral mucosa and in HPV related oral and oropharyngeal cancers are scanty and questionable. Although it is well known that CK19 and/or its fragment CYFRA 21–1 correlate with tumor progression both in the uterine cervix and in the upper aerodigestive tract [31–33] and are statistically significantly connected to patients outcome [34–36], the exact role of CK19 in the genesis of HPV related oral and oropharyngeal cancer requires further assessment [37].

Aims of the present work have been firstly to detect HR-HPV DNA in OSCC/OPSCC, secondly to value the pattern of expression of CK19, and finally to demonstrate the CK19 overexpression in neoplastic cells (compared to the normal areas) in order to prove the possible relationship between HR-HPV^+^ oral/oropharyngeal cancer and the high levels of CK19 expression.

## Results

Our study-cohort was composed of 38 patients (11 females and 27 males), with a mean age of 64.83 years (range:46–89). Characteristics of the patients group are shown in Table 1.

**Table 1:** Baseline characteristics of the 38 patients with OSCC/OPSCC

Variable	Category	N (%)
Age (years)	< 64	18 (47.4)
	≥ 64	20 (52.6)
Sex	Male	27 (71)
	Female	11 (29)
Stage	0	1 (2.6)
	I	6 (15.8)
	II	6 (15.8)
	III	4 (10.5)
	IV	9 (23.7)
	ND	12 (31.6)
Differentiation	Well	5 (13.1)
	Moderate	18 (47.4)
	Poor	15 (39.5)
Site	OSCC (tongue, trigonous, gums and cheeks) /OPSCC (pharynx tonsils)	36 (94.7)
	nodal metastases from OSCC	2 (5.3)
WADA	0 (absent)	9 (23.7)
	1 (focal)	6 (15.8)
	2 (moderate)	4 (10.5)
	3 (intense)	11 (28.9
	4 (MALT)	8 (21.1)
HR-HPV	negative	28 (73.7)
	positive	10 (26.3)

*ND,* *not determined*

The study also included 10 negative control cases of non-neoplastic oral (n.5) and oropharyngeal mucosa (n.5): these specimens were negative for HPV-DNA by ISH assay using Inform HPV family-III (Ventana - Roche) and Inform HPV family-II (Ventana - Roche) and consensus primer PCR.

We have also included some control cases of uterine cervix HR-HPV positive lesions (n.3 cases of High-grade Squamous Intraepithelial Lesion (HSIL)), all of them previously characterized for HPV by PCR followed by direct sequencing.

*Regarding immunohistochemical analysis,* overall, the average expression of CK19 in OSCC/OPSCC was 40.7 % which resulted statistically significant when compared to the corresponding normal, distant and/or peritumoural epithelium (*p* < 0.05). Furthermore, considering the estimated statistical cut-off value of 67 % of stained cells, we have identified two cancer groups: CK19^high^OSCCs/OPSCCs (13 cases) and CK19^low^OSCCs/OPSCCs (25 cases). Considering the staining intensity of CK19 expressing cells, we observed 9 cases scored as 0, 3 cases scored as 1+, 3 cases scored as 2+ and 23 cases with score 3+. On this basis we subdivided OSCCs/OPSCCs in two groups: CK19^faint^OSCCs/OPSCCs (12 cases with score 0–1) and CK19^strong^OSCCs/OPSCCs (26 cases with score 2–3). Moreover, according to the final score (0–300), OSCCs/OPSCCs have been divided in two classes: CK19^high score^OSCCs/OPSCCCs (15 cases with score > 150) and CK19^low score^OSCCs/OPSCCs (23 cases with score < 150). Finally, a wide variability in the intensity and a prevalent distribution in the basal layers have been observed in the controls of normal oral and oropharyngeal mucosa.

Immunohistochemical results were statistically correlated with the clinico-pathological findings (sex, age, tumour site, inflammatory infiltrate surrounding the tumour mass, tumour stage and histological differentiation, peritumoral dysplasia, presence of hyperkeratosis) and evaluated by statistical univariate analysis (ANOVA). No statistically significant correlations have been observed.

*Regarding HPV detection in OSCC/OPSCC,* among 38 cases, 10 were HR-HPV^+^ cancers (as evaluated by ISH and/or consensus PCR) (Table 2): 6 cases were HPV16^+^, 1 case was infected by both HR- and LR-HPVs (HPV31/44^+^), 1 case was HPV31^+^ and 1 case HPV56^+^; the remaining positive case, at the ISH evaluation, showed an integrated status for HR-HPV, but it has not been possible to assess the viral genotype. Among the HR-HPV^+^ cases as resulted by consensus PCR and/or ISH, 6 cases were investigated by ISH also to determine the integrated and/or episomic status of the virus: in 5 cases the HR-HPV was associated to an integrated status; only 1 case resulted negative with cytoplasmic signals, but the HR-HPV positivity was established by consensus PCR. Finally, among the 28 HR-HPV^−^ cancers, 2 were HPV6^+^ (Low Risk- Human Papilloma Virus, LR-HPV), 1 was HPV53^+^ (Intermediate Risk- Human Papillloma Virus, IR-HPV) and the remaining cases (n.25) were HPV^−^.

**Table 2:** HR-HPV detection in OSCC/OPSCC, as evaluated by ISH and/or consensus PCR

Case	Origin	Sex	Age (ys)	ISH	PCR/Sequencing
1	AN	M	74	HR-HPV integrated	HR-HPV 16
2	AN	M	59	HR-HPV integrated	HR-HPV 16
3	AN	F	61	negative	HR-LR-HPV 31-44
4	PA	F	60	ND	HPV 56
5	PA	M	75	ND	HPV 16
6	FG	M	64	ND	HR-HPV 31
7	FG	F	63	HR-HPV integrated	ND
8	FG	F	79	ND	HR-HPV 16
9	NA	M	67	HR-HPV integrated	HR-HPV 16
10	NA	F	69	HR-HPV integrated	HR-HPV 16

**Fig. 1:**
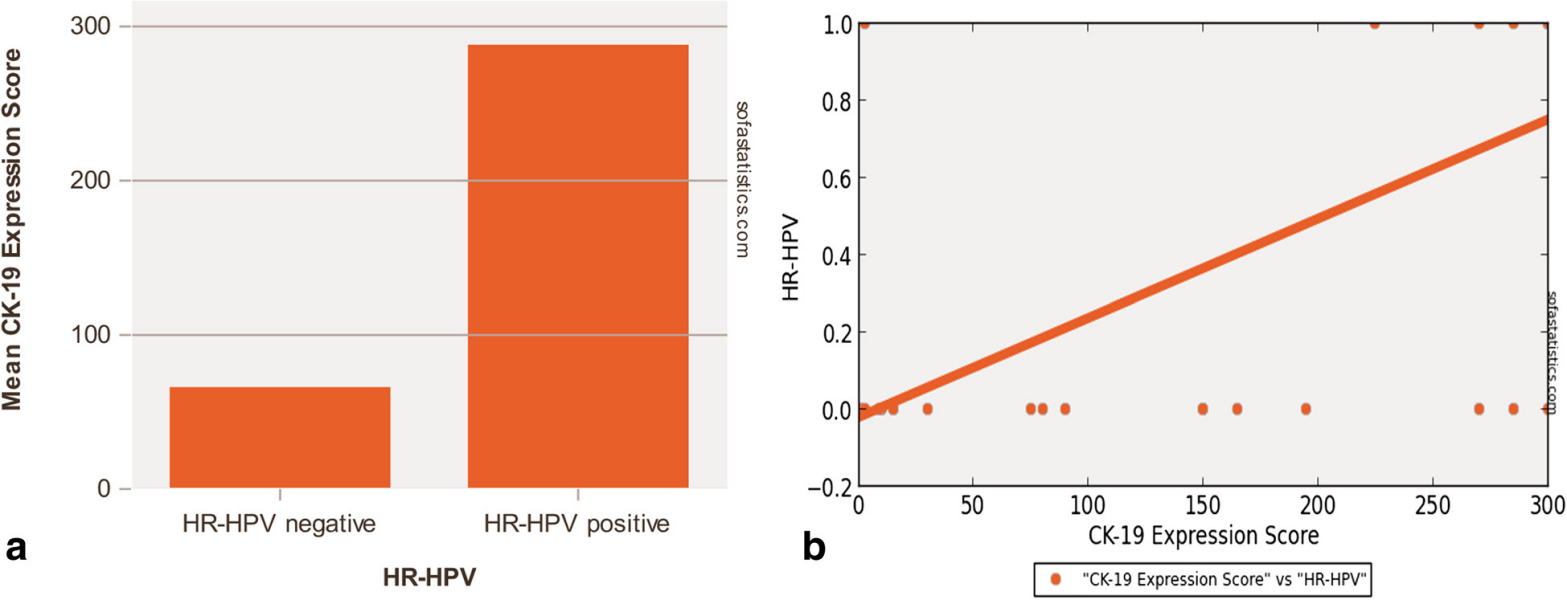
CK19 expression score in HR-HPV^+^ and HR-HPV^−^ OSCCs/OPSCCs. **a**. Differences in CK19 expression scores according to HR-HPV infectious status. **b**. ROC curve with a strong evidence of correlation between HR-HPV positive result and CK19 expression (Sensitivity: 92.3 %; Specificity: 89.3 %; *p* <0.001) and (*p* <0.001; Spearman’s R: +0.72)

**Fig. 2:**
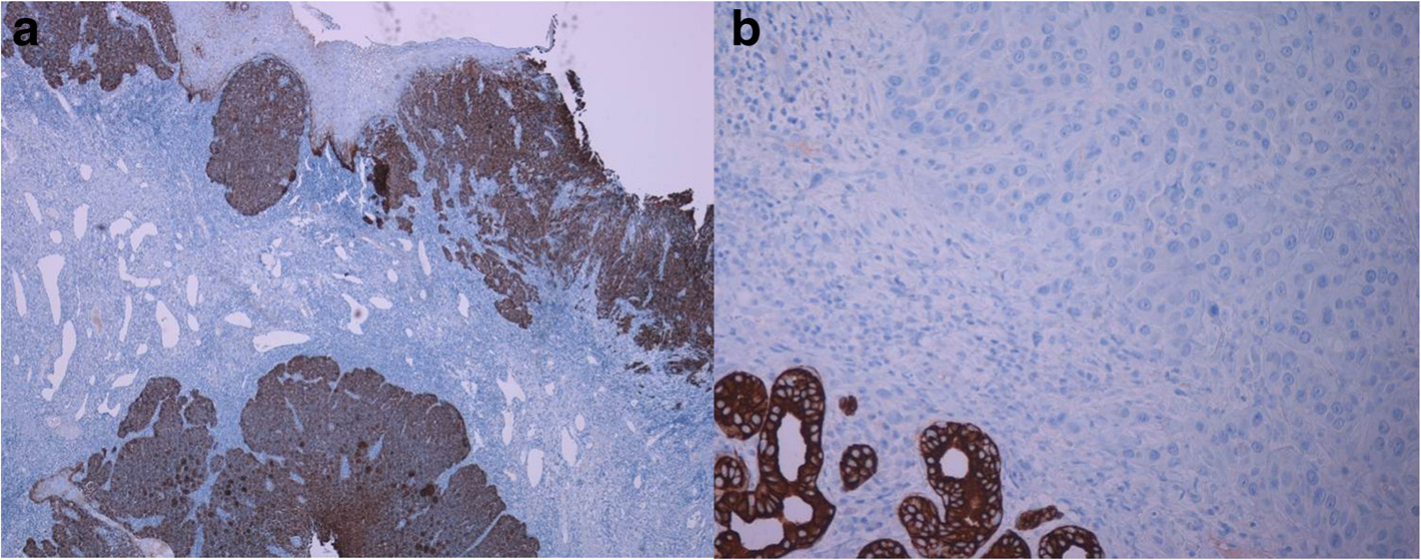
Immunohistochemical expression of CK19 in HPV^+^ and HPV^−^ OSCCs. **a**. Note the strong and diffuse immuostaining for CK19 in a representative case of HPV related OSCC. **b**. A complete CK19 negative staining in HPV negative OSCC can be observed. Internal control is represented by the positive staining for CK19 in salivary glands. (LSAB-HRP, nuclear counterstaining with haematoxylin; original magnification **a**, x40; **b**, x100)

*Regarding the correlation between CK19 expression and HR-HPV infection in OSCC/OPSCC,* we have noted that CK19 expression scores were very different in the two groups of analyzed cancers (HR-HPV^+^/HR-HPV^−^). We obtained higher values in HR-HPV positive group (mean: 288.0 ± 24.3) than in negative one (mean: 66.2 ± 96.9) (Figs. 1a and 2; Table 3). This difference was statistically significant (*p* < 0.001) with a strong evidence of correlation (*p* < 0.001; Spearman’s R: +0.72). We described ROC curve with a cut-off > 195 for HR-HPV positive result (Sensitivity: 92.3 %; Specificity: 89.3 %; *p* < 0.001) (Fig. 1b). High scores (270–300), high percentages (90–100) of expressing cancer cells and a constant staining 3+ have been observed in all integrated HR-HPV^+^ OSCCs/OPSCCs.

**Table 3:** Comparison between HR-HPV negative vs HR-HPV positive OSCCs/OPSCCs and CK19 expression score (Independent Samples *t*-test) *p* value: < 0.001

Group	*N*	Mean	CI 95 %	Standard deviation	Min	Max
HR-HPV negative	28	66.214	30.332 - 102.097	96.873	0.0	300.0
HR-HPV positive	10	288.0	272.945 - 303.055	24.290	225.0	300.0

Heterogeneous areas of immunoreactivity varying in percentage value, intensity and/or localization were observed in normal epithelium, both perilesional and distant from the tumor.

*In perilesional areas, in HR-HPV*^*−*^*cancers,* we observed CK19 positivity in 13 out 19 valuable cases (68.4 %) and a generally moderate-strong staining, localized in all epithelial layers (6 cases at basal level, 4 cases at the intermediate layer and the remaining 3 cases in the upper layer).

*In HR-HPV*^*+*^*cancers* we observed a constant expression of CK19 (positivity in 7 out 7 valuable cases; 100 %) and a generally moderate-strong staining, mostly localized to the upper layers.

**Fig. 3:**
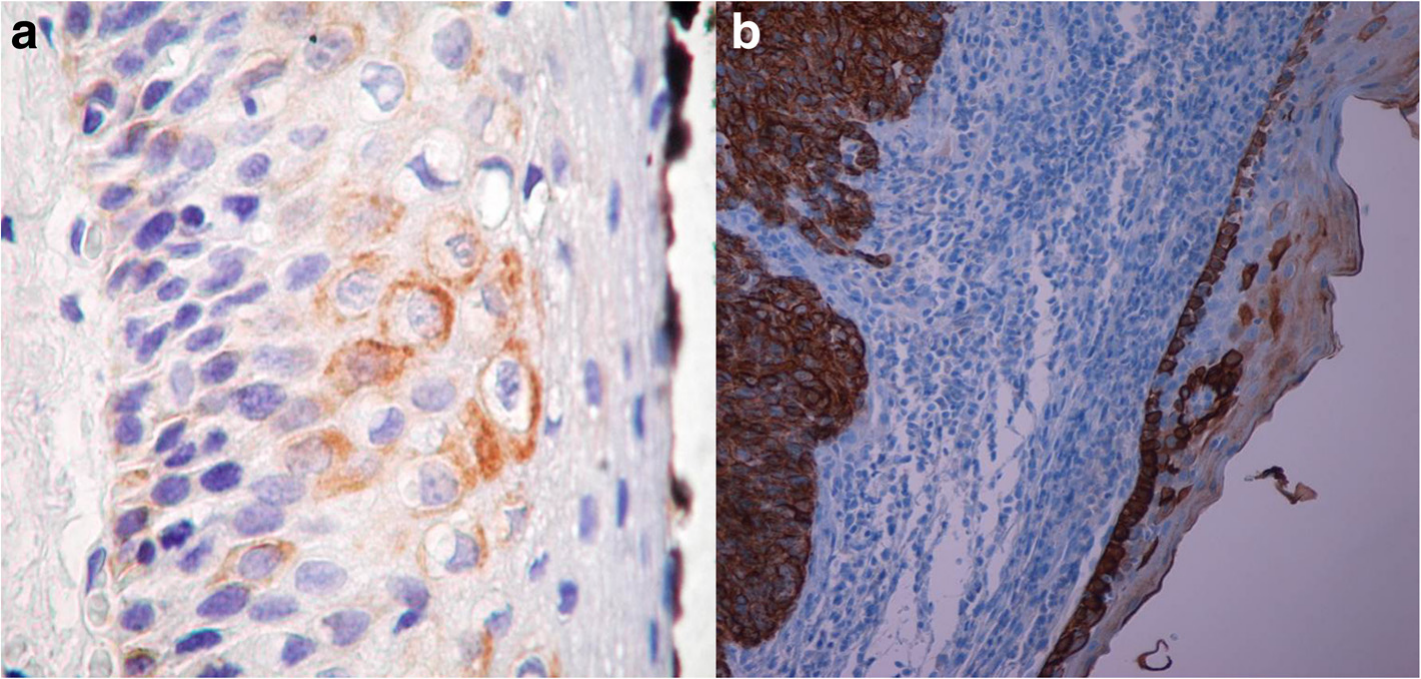
CK19 staining expression and localization in perilesional mucosa in HPV^−^/HPV^+^ cancers. **a**. CK19 expression is evident in basal and intermediate layer in dysplasia surrounding HPV^−^OSCC. **b**. Strong expression of CK19 in a basaloid OPSCC. Note the overhead mucosa with CK19 up-regulation and maturation disturbance. (LSAB-HRP, nuclear counterstaining with haematoxylin; original magnification **a**, x200; **b**, x40)

The differences regarding CK19 staining localization were statistically significant (*p* = 0.017) (Fig. 3, Table 4). No statistical significance has been observed regarding the difference in intensity of CK19 staining.

**Table 4:** Comparison between HR-HPV negative vs HR-HPV positive OSCCs/OPSCCs and CK19 staining expression and localization in perilesional mucosa (Mann Whitney *U* Test) Two-tailed *p* value: 0.017

Group	N	Median	Avg Rank	Min	Max
HR-HPV negative	19	1.0	11.368	0.0	3.0
HR-HPV positive	7	3.0	19.286	1.0	3.0

*In mucosal areas distant from OSCCs/OPSCCs, in HR*-*HPV*^*−*^*cancers* we observed a prevalent absence of CK19 expression (58.9 %) and when present (n. 7 out 17 valuable cases; 41.1 %), we noted a wide variability in the intensity of CK19 staining, distributed mostly in the basal layers.

**Fig. 4:**
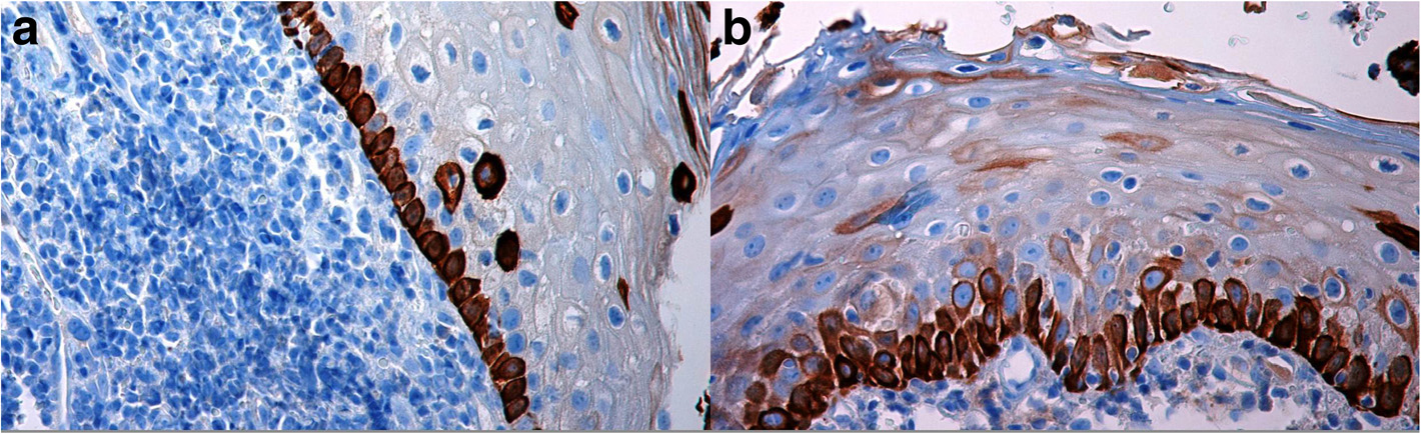
CK19 staining expression and localization in distant mucosa in HPV^−^/HPV^+^ cancers. **a**. CK19 expression was distributed mostly in the basal layers in distant mucosa from HPV^−^ OSCC. **b**. CK19 staining was generally moderate in intensity, localized both at basal and more superficial epithelial layers in distant mucosa from HPV^+^ OSCC. (LSAB-HRP, nuclear counterstaining with haematoxylin; original magnification **a**, x200; **b**, x200)

*In HR-HPV*^*+*^*OSCCs* CK19 staining was always present (positivity in 4 out 4 valuable cases; 100 %), generally moderate-strong in intensity, localized equally at basal and superficial epithelial layers. The differences regarding CK19 staining intensity (Table 5A) and localization (Fig. 4, Table 5B) were statistically significant (*p* = 0.032 for the intensity; *p* = 0.025 for the localization).

**Table 5:** Comparison between HR-HPV negative vs HR-HPV positive OSCCs/OPSCCs, CK19 staining expression (intensity in A, localization in B) in distant mucosa (Mann Whitney *U* Test)

5A) Two-tailed *p* value: 0.032					
Group	*N*	Median	Avg Rank	Min	Max
HR-HPV negative	17	0.0	9.647	0.0	3.0
HR-HPV positive	4	3.0	16.75	2.0	3.0
5B) Two-tailed *p* value: 0.025					
Group	*N*	Median	Avg Rank	Min	Max
HR-HPV negative	17	0.0	9.588	0.0	3.0
HR-HPV positive	4	2.0	17.0	1.0	3.0

**Fig. 5:**
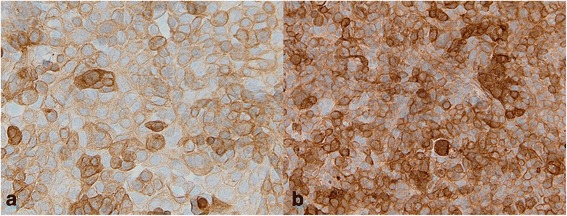
Immunocytochemical expression of CK19 in HPV^−^ OSCC cell line (SCC-131) and in HPV^+^ OSCC cell line (SCC-154). The two cell lines were both positive to CK19 antibody but showed a different level of expression. **a**. Note that the UPCI-SCC-131 cell line showed a lower level of expression, with mostly moderate, focally strong intensity of the staining. **b**. UPCI-SCC-154 cell line had a higher level of expression, characterized by strong intensity and a diffuse pattern. (LSAB-HRP, nuclear counterstaining with haematoxylin; original magnification, x200)

Finally, we measured the variation of CK19 expression in UPCI-SCC-131 and UPCI-SCC-154 cell lines by ICC and flow cytometry. The two cell lines were both positive to CK19 antibody but showed a different level of expression, in particular it should be noted that the UPCI-SCC-154 cell line had a higher level of expression, characterized by strong intensity and a diffuse pattern; on the other hand, UPCI-SCC-131 cell line showed a lower level of expression, with mostly moderate, focally strong intensity of the staining (Fig. 5).

**Fig. 6:**
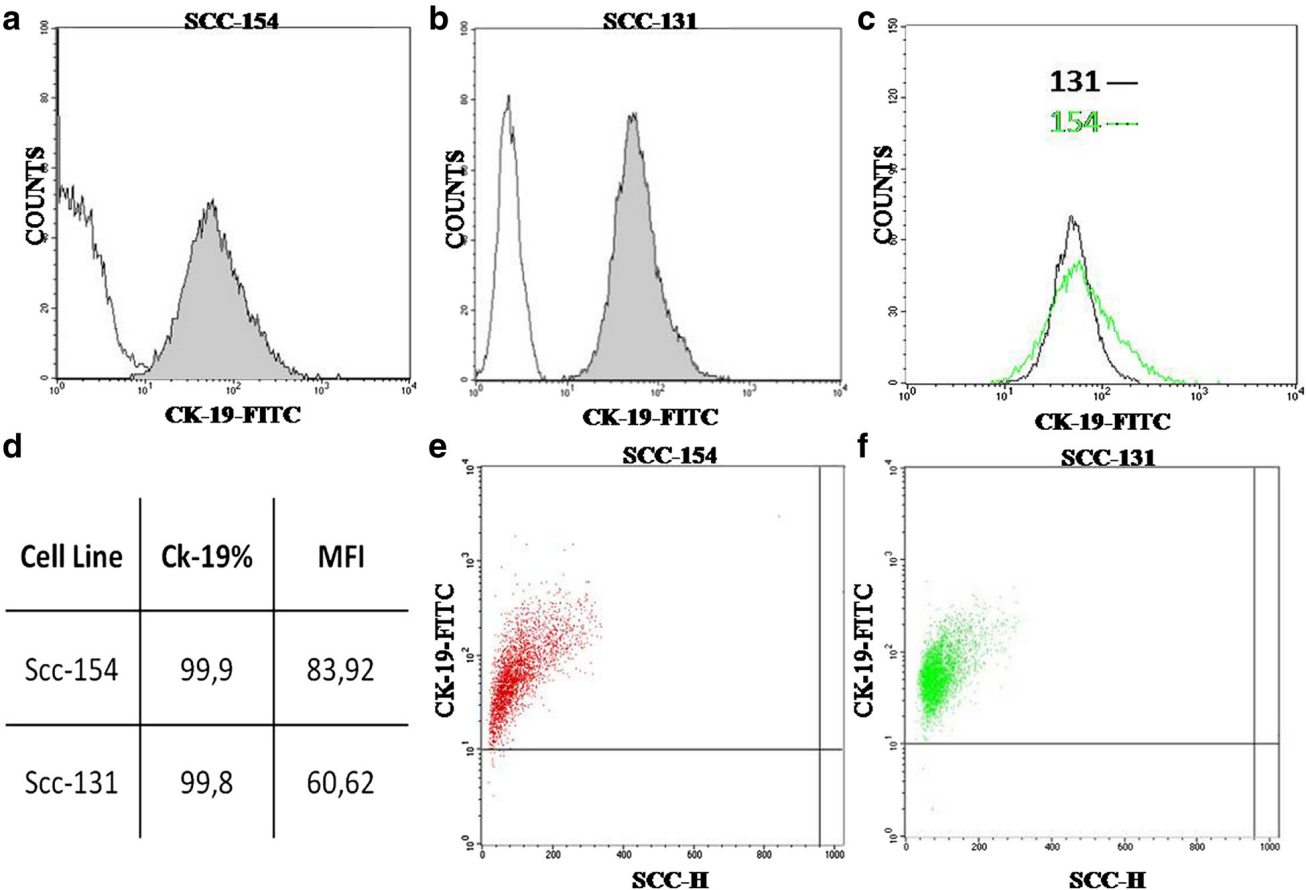
Representative FACS analysis. Histograms for SCC-154, and SCC-131. **a**, **b** Sample treated with antibody to CK19 conjugated with FITC and with relative isotope control. **c** Overlay of Histograms. Fluorescence values on a logarithmic scale are shown on the x-axis and counts cells are scored on the y-axis. **d** Table summarizing the differential CK19 expression on SCC cell lines. The CK19 percentage represents the fraction of cells that linked the FITC-conjugated CK19 antibody, as compared to the total analyzed cell population. The mean fluorescence intensity (MFI) per cell has also been reported. **e**-**f**) The plot of cell physical parameter (SCC versus CK19-FITC)

UPCI-SCC-131 and UPCI-SCC-154 cell lines were also characterized for intracellular expression of CK19 by flow cytometry analysis. The cytofluorimetric pattern of CK19 expression is well illustrated in Fig. 6. From the flow cytometry histograms resulting from the two cell lines, stained with the fluorescent antibody against CK19 with relative isotope control (Fig. 6a for UPCI-SCC-154, Fig. 6b for UPCI-SCC-131 and from the overlay of the two previous histograms (Fig. 6c), we have demonstrated that the cell line UPCI-SCC-154 (HPV^+^) had a greater fluorescence value. Although the two studied cell lines were both positive for CK19 expression, different rates of positivity have been reported. The relative extent of CK19 expression, as revealed by the mean fluorescence intensity (MFI), varied among the analyzed cell lines, being lower for UPCI-SCC-131 (MFI = 60,62) in comparison to the UPCI-SCC-154 (MFI = 83,92) (Fig. 6d). The absolute cell counting was performed on SSC/CK19 dot plots (Fig. 6e-f): both cell lines were positive for CK19.

## Discussion

Since the HR-HPV subtypes are always more frequently linked to aetiology of many human cancers including oral and oropharyngeal cancer, an innovative approach to the study of OSCC/OPSCC should be based on a better understanding of the molecular viral background and the relative interaction between virus and host cell. It has well established that some HR-HPVs (in particular HPV16) induce immortalization of keratinocytes and at the same time the deregulation of the normal CK expression pattern in stratified squamous epithelium [22]. The investigation of CKs expression profile in HPV-related OSCCs/OPSCCs and the study of its potential value as possible predictor of viral infection and neoplastic progression could allow to characterize a possible evolutive morphologycal profile of malignancies.

Cytokeratin 19 (CK19), a 40 kDa acidic cytokeratin (Type I), is normally expressed in stratified squamous epithelium and it has been considered a marker of pre-malignancy and susceptibility to cancers like the OSCC [38].

Although we have not observed any type of correlation between immunohistochemical CK19 expression and the analyzed clinico-pathological findings, a recent study has confirmed CK19 as a valid prognostic marker in human OSCC and its over-expression as an important molecular event in pathogenesis of oral carcinoma [39].

Beforehand and similarly, other Authors have evaluated the role of CK19 in OSCC, reporting different percentages of expression [40–42].

In our study, considering an average CK19 expression in cancer of 40.7 % and an estimated statistical cut-off value of 67 % of stained cells, we have identified two cancer groups: CK19^high^OSCCs/OPSCCs and CK19^low^OSCCs/OPSCCs. According to *staining intensity*, generally, it was mainly moderate or strong (3 cases scored as 2+ and 23 cases with score 3+).

Heterogeneous areas of immunoreactivity varying in percentage value, intensity and/or localization were also observed in normal epithelium, both perilesional and distant from the tumor.

In perilesional areas, we mostly observed a moderate-strong positivity for CK19, distributed in all epithelial layers (mostly at basal layer) and with lower percentages in HR-HPV^−^ cancers than in HR-HPV^+^ ones (68.4 % vs 100 %) where the marker preferentially was located to the upper layers. In more distant areas, in HR-HPV^−^ OSCCs/OPSCCs CK19 staining was observed in 41.1 % of cases with a wide variability in the intensity and distributed mostly in the basal layers, as observed in normal oral/oropharyngeal mucosa; differently, in HR-HPV^+^ OSCCs/OPSCCs CK19 was present in 100 % of cases, generally moderate-strong in intensity and localized in all epithelial layers.

Considering its low molecular weight, CK19 in normal conditions should be mostly located in the basal/parabasal layers [43]. In our sample in the perilesional areas, in areas involved by preneoplastic changes, and also in areas distant from the tumor but in the context of an HR-HPV infected mucosa, as morphological sign of an occurred disturb in the normal differentiation program, we have observed that CK19 was distributed equally in all epithelial layers, from the basal to the superficial ones or preferentially in the upper levels.

Already in the past, it was suggested that when CK19 appears in the suprabasal cell layers in oral epithelium, it might be a consequence of a delay or a disturbance in the terminal differentiation, probably indicating retention of hyper-proliferative potential and acquisition of cellular atypia associated to premalignancy [44]. On the other hand, according to the idea that oral mucosa, as well as genital cervix mucosa, is a target for frequent frictions and irritations, thus needing a more rapid epithelium regeneration, other Authors, since the far 90’s, underlined that keratinocytes normally contain CK19 also in suprabasal layers [30, 45].

Anyway, it still remains to understand the exact role of CK19 in the genesis of HPV related oral and oropharyngeal cancer and how keratinocyte differentiation could be involved in pathogenesis of HPV infection [46]. In 1991 Kellokoski J et al. observed a statistically significant difference between HPV-DNA and HPV-DNA negative oral biopsies regarding CK19 staining, being the last more intense in virus associated samples. They interpreted their results suggesting that viral infection could modify the keratinization of oral mucosa, disturb the keratinocyte differentiation and exert proliferative potential in basal cells, thus accelerating the production of low molecular weight cytoskeletal protein [30]. From 1991 to nowadays, a long time of scientific silence about the relationship between HPV and CK19 expression is passed. In 2004 in a study performed on two cancer cell lines (one derived from cervical squamous carcinoma, the other one from a metastatic site of cervical carcinoma), Favia et al. [47] have analyzed the interaction occurring between HPV type 16 E7 mRNA and the intermediate cytokeratin filaments 7 and 19 and reported data in favor of a possible association between HPV16 E7 protein level and CK19. The same Authors have highlighted the opposite effect of cytokeratins 7 and 19 on HPV16 E7 oncoprotein expression, with CK7 involved in protecting and storing the E7 transcript and CK19 assuring the viral mRNA translation into the oncogenic product and, in turn, leading to the possibly related carcinogenic events [48].

## Conclusions

Considering that HPV unrelated OSCCs/OPSCCs are generally quite resistant to chemotherapeutic agents while HPV related cancers are characterized by radio-chemo sensitivity, HPV detection and in particular HR-HPV identification are of basic importance in HNSCC patients clinical management. Up to now, in spite of the significant number and the types of molecular available methods or their combinations, there is not a general recognized agreement about the ‘golden standard’ identification test.

The currently performed techniques of viral detection in clinical practice such as ISH, routinely IHC for viral oncoprotein (E5-6-7) and p16-IHC test [16, 49] are considered not satisfactory when evaluated as HPV detecting tests as used alone, for their low sensitivity [50, 51], the limited antibodies availability (IHC), the low applicability in clinical routine (ISH) and the low specificity (p16). In particular, p16-IHC as diagnostic method has caused much debate [52], since p16 over-expression might be associated with functional pRb alterations that are independent from the HPV infection [53] and even not all HR-HPV-infected OPSCCs have lost the 9p21 allele encoding p16 [54].

Recently, in order to distinguish HPV^+^ versus HPV^−^ OSCCs/OPSCCs innovative techniques, as well as SPF10 HPV DNA test, PGMY/GP nested PCR system, quantitative E6 RNA PCR [55, 56] and c-DNA microarrays [57, 58], are merging and could be considered highly sensitive molecular screening test with a very high level of accuracy.

In an our previous study we have clearly demonstrated that a combined triple method, consisting in p16-IHC/ISH/Consensus PCR test, preserves the morphological context of HPV-DNA signals in formalin-fixed, paraffin-embedded (FFPE) samples and increases the overall specificity and sensitivity in HPV detection [59].

The presented study adds CK19 as a new interesting marker able to determine a differential diagnosis among HPV^+^ and HPV^−^ OSCCs/OPSCCs. We have reported higher values of CK19 expression in HR-HPV^+^ cancers (CK19^high^OSCCs/OPSCCs; mean score: 288.0 ± 24.3) than in negative ones (CK19^low^OSCCs; mean score: 66.2 ± 96.9). This statistically significant difference (*p* < 0.001) suggests a possible association between HR-HPV and CK19 expression.

Similar results have been obtained also by immunocytochemical analyses performed on two SCC-cell lines (differing for the HPV infectious status) and by flow cytometry. Finally, these observations can be regarded as a possible evidence of an interplay existing between HPV oncogenic activity and keratinocyte differentiation.

We conclude that the routinely evaluation of an immunohistochemical panel including p16, and CK19 should be assessed by further investigations since these markers, exclusively and in combination, could add useful informations concerning oral and oropharyngeal carcinogenesis and improve the current diagnostic tools, allowing to characterize a possible clinic-morphologycal profile of oral/oropharyngeal cancer and its precancerous lesions.

## Materials and methods

### Study population

Upon approval by the Ethical Committee of the University of Foggia, 38 subjects affected from OSCC/OPSCC were randomly selected from 4 different Hospitals of the Middle and South of Italy and enrolled in this study (Institute of Pathological Anatomy, University of Foggia; Department of Clinic Specialistic and Stomatological Sciences, Polytechnic University of Marche, Ancona; Institute of Oral Pathology, University of Napoli ‘Federico II’; Department of Stomatological Sciences, University of Palermo). All patients or their relatives gave their informed written consent.

The cases have been randomly chosen from cohort known for the HPV prevalence as previously published [59–61] and were analyzed using FFPE materials coming from surgery with curative intention; in addition, some cases have been also analyzed using byoptical material obtained in preoperative setting. All slides were reviewed by 2 pathologists (ASanto, GP) at the Institute of Pathological Anatomy, University of Foggia, Foggia, Italy. Tumour stage and grade were assigned according to the WHO classification of malignant tumours of the head and neck [62].

Finally, we have also selected an adequate group of non-neoplastic oral (n.5) and oropharyngeal mucosa (n.5) samples and some control cases of uterine cervix HR-HPV HSILs (n.3) for the comparative statistical evaluations. Characteristics of the group of the patients are shown in Table 1, where we have reported clinical data referring to patients’ sex, age, and histological characteristics of the tumors (such as site, TNM staging, grading, infectious status). We have also assessed the WADA grade, defined as the entity and the distribution of inflammatory cells in the lamina propria and in the submucosal tissue [63].

Finally, at the Institute of Pathological Anatomy, University of Foggia, a total of 38 archival FFPE OSCCs/OPSCCs were investigated by standard IHC for CK19, by consensus HPV-DNA PCR methods to detect HPV infection and by ISH to study the viral integration status into the host DNA.

### IHC

IHC was performed on 4 μm paraffin sections mounted on poly-L-lysine-coated glass slides, by standard linked streptavidin-biotin horseradish peroxidase (LSAB-HRP) technique, using a specific monoclonal antibody against CK19 (mAb clone A53-B/A2.26), by the Benchmark XT autostainer (Ventana Medical Systems Inc, Tucson, AZ). Cases were evaluated on the basis of percentage of positive cells and, considering a statistical cut-off of 67 %, they were distinguished into two categories: CK19^high^OSCCs/OPSCCs and CK19^low^OSCCs/OPSCCs. Then, we have also evaluated the staining intensity, that has been scored as follows: negative (0), faint (1), moderate (2), strong (3). Finally, intensity staining has been multiplied to IHC percentage in order to obtain the final molecular expression score, ranging from zero to 300 units. Appropriate positive and negative controls were run for the tested antibody. On our OSCC/OPSCC samples, serial sections, in addition to the neoplastic area, also included perilesional areas and mucosal areas > 5 cm distant from the tumor. Moreover, negative controls were performed on other sections that comprised normal areas of removed oral and oropharyngeal mucosa for surgical non-neoplastic diseases; negative control slides without primary antibody were also included. Positive control was executed on sections obtained from a case of infiltrating colon cancer. Inter-rate reliability between the two investigators blindly and independently examining the immunostained sections was assessed by the Cohen’s K test, yielding K values higher than 0.7.0 in almost all instances.

### PCR analysis

HPV DNA was researched by nested PCR (MY/GP primers), and HPV genotype was determined by direct sequencing of PCR fragments. Three types of control were included in each reaction series: blank control, HPV-neg.ve Wi cells as neg.ve control and HPV18 DNA-pos.ve HeLa cells, in dilutions from 20,000–50,000 down to 2–5 HPV DNA copies, as pos.ve control. HPV DNA was amplified by PCR assay using primers useful for samples with a low copy number of HPV (MY09–MY11 primer pair in combination with GP5–GP6 primer pair) as previously demonstrated in current literature [64] and amplifications were performed in a DNA thermal cycler (Mastercycler gradient; Eppendorf, Hamburg, Germany); amplification products were analyzed in 8 % polyacrylamide gel.

### Sequencing analysis

HPV genotyping was based on direct sequencing of MY or MY/GP PCR fragments. Amplification products were purified by Microcon YM-100 (Amicon-Millipore, Billerica, MA); the sequence of both DNA strands was determined by the BigDye Ready Reaction Kit in the automatic sequencer ABI Prism 310 Analyzer (both from Perkin-Elmer Applied Biosystems, Foster City, CA). Alignments were obtained from the GenBank on-line BLAST server and HPV sequences downloaded from the HPV database (http://www.ijbcb.org/HPV/).

### ISH for HPV DNA detection

ISH has been performed using the Benckmark® XT plate and an indirect alkalin-phospathase antibody mediated detection method. The hybridization signals were showed with Tetrazole Blu and Fast Red nuclear counterstaining. The commercially available Ventana kit includes the following probes for HR-HPV: 16, 18, 31, 33, 35, 39, 45, 51, 52, 56, 58, e 66 (INFORM HPV III Family 16 Probe; Ventana – Roche); and the following probes for LR-HPV: 6, 11 (Inform HPV Famly II 6 Probe; Ventana – Roche).

### ISH evaluation

ISH signals have been evaluated on at least ten High power fields at Olympus BX-41 optical microscope (High Power Field, HPF, original magnification x 40). OSCC/OPSCC cases showing prominent nuclear punctuated (discreet dot-like) signals have been considered as integrative (I). Cases with exclusive nuclear cluster signals as been evaluated as episomal (E). Cases showing a prevalent nuclear cluster signals along with also focal punctuated signals of integration have been evaluated as mixed episomic-integrative (E-I). According to manufacturer instructions, artifacts or non-specific findings as been considered the followings: non-cellular stromal precipitates; cytoplasms of polymorphonuclear leukocytes (PMNs), eosinophils, lymphocytes and endothelial cells; non-specific staining of nucleoli.

### Cell culture and treatment

Cell lines derived from OSCCs and normal keratinocytes have been used for ICC and cytofluorimetric analyses. The cell lines used were (1) UPCI-SCC-131 (originated from a human oral squamous cell carcinoma of a 73-year-old Caucasian man) (2) UPCI-SCC-154 (HPV^+^) (originated from a human oral squamous cell carcinoma of a 54-year-old Caucasian man). The cell lines were purchased from the DSMZ, Braunschweig Germany.

The cell lines were grown in 90 % minimum essential medium (MEM with Earle’s salts) supplemented with non-essential amino acids, 10 % fetal bovine serum (FBS), 2 mM L-glutamine,1 % penicillin and 1 % streptomycin in a humidified incubator containing 5 % CO2 at 37 °C.

### ICC

Cells were grown on four-chamber tissue culture-treated glass slides (Falcon Becton Dickinson, Labware, NJ, USA) pre-coated with poly-L-lysine to enhance cell attachment at a density of 5000 cells per well. Cultured cells were rinsed with PBS, and finally fixed in alcohol. Immunostaining was performed using LSAB-HRP technique and specific mouse monoclonal Ab anti CK19 (mAb clone A53-B/A2.26). Evaluations of the immunocytochemical staining were performed separately by two observers with at least 10 HPFs using optical microscopy.

### Flow cytometry

To detect CK19 nonconfluent cultures were trypsinized into single cell suspension, counted, washed with phosphate-buffered saline (PBS), fixation and permeabilization by intracell kit (immunostep, S.L. Avda). Then the cells were incubated with FITC conjugated CK19 antibody ab87014 or isotope antibody ab81197 (both from Abcam) as negative control for 1 h. After washed twice with PBS, samples were analyzed by FACSCalibur (Becton Dickinson).

### Statistical analysis

All data have been analyzed by MedCalc 12.2.1.0 (for Windows), SOFA Statistics 1.4.3 and R 2.11.1 (for Linux) statistical softwares using Debian 7 and Windows Operating Systems.

Differences between groups were determined using the Independent Samples *t*-test of average. Spearman’s method and Point-biserial correlation coefficient were used to study linear correlation and to determine the relationship between CK19 expression and HR-HPV positivity (1 = HR-HPV integration positive signals; 0 = negative ISH for HR-HPV). In order to select a relevant CK19 cut-off score to identify HPV positive samples, Receiver Operating Characteristic (ROC) curve analysis was carried out. The point on the curve, maximizing sensitivity and specificity of CK19 expression score, was selected as the cut-off score above which it was considered positive marker of HPV positivity. The expected average value of the ROC area is 0.5 if there is no discrimination between the groups. In order to distinguish a real discrimination between the groups from the case of no discrimination, a *p*-value was calculated. A small *p*-value makes it unlikely that the ROC area can be reconciled with the case of no discrimination. Only values of *p* < 0.01 were considered significant.

## Abbreviations

HR-HPV: High risk-human papilloma virus
OSCCs: Oral squamous cell carcinoma
OPSCCs: Oro-pharyngeal squamous cell carcinoma
IHC: Immunohistochemistry
Ab: Antibody
ISH: In Situ Hybridization
PCR: Polymerase chain reaction
ICC: Immunocytochemistry
HNSCCs: Head and neck squamous cell cancers
CK: Cytokeratin
HMW-CKs: High molecular weight cytokeratins
LMW-CKs: Low molecular weight cytokeratins
LR-HPV: Low risk-human papilloma virus
IR-HPV: Intermediate risk-human papilloma virus
HSIL: High-grade squamous intraepithelial lesion
FFPE: Formalin fixed paraffin embedded
LSAB-HRP: Linked StreptAvidin-Biotin HorseRadish Peroxidase
HPF: High power field
I: Integrative
E: Episomic
E-I: Episomic-integrative
PMN: Polymorphonuclear leukocytes
PBS: Phosphate-buffered saline
ROC: Receiver operating characteristic
MFI: Mean fluorescence intensity
